# Microstructural Origins of Nonlinear Response in Associating Polymers under Oscillatory Shear

**DOI:** 10.3390/polym9110556

**Published:** 2017-10-26

**Authors:** Mark A. Wilson, Arlette R. C. Baljon

**Affiliations:** 1 Computational Materials and Data Science, Sandia National Laboratories, Albuquerque, NM 87123, USA; marwils@sandia.gov; 2 Department of Physics, San Diego State University, San Diego, CA 92182, USA

**Keywords:** associating polymers, oscillatory shear, MD/MC simulations, nonlinear response

## Abstract

The response of associating polymers with oscillatory shear is studied through large-scale simulations. A hybrid molecular dynamics (MD), Monte Carlo (MC) algorithm is employed. Polymer chains are modeled as a coarse-grained bead-spring system. Functionalized end groups, at both ends of the polymer chains, can form reversible bonds according to MC rules. Stress-strain curves show nonlinearities indicated by a non-ellipsoidal shape. We consider two types of nonlinearities. Type I occurs at a strain amplitude much larger than one, type II at a frequency at which the elastic storage modulus dominates the viscous loss modulus. In this last case, the network topology resembles that of the system at rest. The reversible bonds are broken and chains stretch when the system moves away from the zero-strain position. For type I, the chains relax and the number of reversible bonds peaks when the system is near an extreme of the motion. During the movement to the other extreme of the cycle, first a stress overshoot occurs, then a yield accompanied by shear-banding. Finally, the network restructures. Interestingly, the system periodically restores bonds between the same associating groups. Even though major restructuring occurs, the system remembers previous network topologies.

## 1. Introduction

In most areas of soft matter research, it is safe to think of chemical bonds as an entity that changes little over time. Not so in polymer gels, where “dynamical bonds” or “sticky junctions” continuously undergo reversible breaking and reformation. These gels typically consist of associating polymers that contain hydrophobic or ionic groups with more affinity to each other than to the polymeric backbone. They are incompatible with the solvent and hence form aggregates [1,2,3]. The number of reversible bonds formed is conditioned by external parameters such as temperature, concentration, and mechanical stress. If this number reaches a critical value, gelation occurs and a space-spanning network forms. Due to the reversible nature of the bonding, the system still restructures continually even in the gel state. For dynamical arrest, a much higher number of bonds than the critical value is required.

Associating polymers can add unique mechanical characteristics to materials, desirable for a multitude of applications [4]. They have a long-standing use in industrial applications as rheological modifiers. Their general fluid-thickening characteristics have made them a regular additive to paints, adhesives, and sealants [5]. Applications in the medical field of late have raised new interest. Chemo-responsive gels, for instance, are synthetic materials that make use of reversible chemistry to acquire properties heretofore only found in living matter. They are developed for use as artificial muscle and in tissue engineering [6,7]. Also explored are novel network-based techniques to study collagen fiber organization and reorganization induced by external loads [8].

Linear viscoelastic properties of associating polymer networks have been studied extensively in the literature, typically by applying a small amplitude oscillatory shear (SAOS). If the lifetime of sticky junctions is long enough, and oscillatory shear is at high enough frequencies, gelation occurs [9]. In order for the system to relax, it needs to break these sticky junctions and reform them between different associating groups [10]. Details of this mechanism have been studied for decades [11,12], but much remains to be resolved. This holds in particular for nonlinear stress response during large amplitude oscillatory shear experiments (LAOS). Besides interactions between the associating groups, overall network topology and history of deformation dictate the emergent properties of these materials. Computational studies have the ability to investigate these, since they can obtain details of the microscopic structural and dynamical changes that underlay the macroscopic rheological response.

Various groups [13,14] have proposed new theories that extrapolate viscoelastic theory from the small-amplitude low-frequency linear to the nonlinear LAOS regime. The earliest proposals in this vein use Fourier transforms to calculate the contributions of higher order harmonics. More recent theories employ Chebyshev polynomials to construct general viscous and elastic moduli that extend the loss and storage moduli to the nonlinear regime. All these theories assume that only the applied strain affects the microstructure and hence these theories average the stresses observed at the same strain. In other words, it is assumed that the system can sufficiently respond to deformation such that its history is irrelevant. Rogers et al. [15,16,17] have argued that this assumption is mistaken. LAOS signals in soft matter, they point out, should be interpreted as the result of a sequence of physical processes—elastic extension, yielding, flow, and reformation. The data presented in this manuscript confirm these processes. They further indicate that shear-induced, microstructural changes, responsible for the nonlinear rheology of the model telechelic polymer system, depend not only on the current strain but also on shear history.

This article complements our recent publication of properties of telechelic associating polymer networks under large amplitude oscillatory shear [18]. That study focused on a comparison of macroscopic properties to those discussed in the literature [14,19]. Strain rate-frequency superposition was performed to overlap the storage and loss moduli (obtained from the primary harmonics). This superposition, pioneered by Wyss et al. [20], follows from the notion that most relaxation processes are dictated by the strain rate rather than by frequency and amplitude. More recently, Hess et al. [21] have argued that instead of overlapping storage and loss moduli, it is better to overlap general moduli in the highly nonlinear domains encountered under LAOS. In either case, it is found that strain rate dominates relaxations for all but the highest frequencies (Rouse regime).

In the manuscript at hand, we investigate in detail two amplitude/frequency combinations at which the response shows the largest deviation from linear behavior. The first combination occurs at a very large strain amplitude (3.59). We attribute its behavior to the significant restructuring needed to accommodate the large displacements (type I). The second combination occurs at a higher frequency, at the point where the elastic storage modulus starts to dominate the viscous loss modulus. Nonlinear collective effects are held responsible for this phenomenon (type II). Both combinations yield the same strain rate. In our previous study [18], we showed the system’s response as a function of frequency at six different strain rates. We found that nonlinearities have the same qualitative features at all strain rates. Type I requires a high strain amplitude—roughly 1.5 and up. Type II tends to occur when the elastic modulus is larger than the viscous one. We believe that microscopic details underlying the two types of nonlinear response that we investigate in the text below are relevant for other amplitude/frequency combinations and strain rates as long as they show a similar stress response.

Since we are interested in the fundamental mechanisms that give rise to nonlinear rheology, we utilize short, unstructured, bead-spring models for computational efficiency. Hence, our results match experimental observations only qualitatively. Given transient phenomena and the need to access low frequencies, some of our data required computer runs of several months using this limited toy model. We believe, nevertheless, that the insight we obtain on how microscopic dynamics of the system gives rise to its overall macroscopic properties might be useful to the interpretation of experiments. In the conclusions, we will point out the qualitative similarities between the nonlinearities of our systems and those reported by others.

## 2. Simulation Method

### 2.1. Molecular Dynamics/Monte Carlo Code

We employ a modified molecular dynamics (MD) simulation based upon the bead-spring framework established by Kremer et al. [22]. In these simulations, polymer chains are modeled as strings of spherical beads, which interact with each other through Lennard-Jones (LJ) potentials:

(1) $$U_{LJ}\left(r_{ij}\right)=\;4\varepsilon \left[{\left(\frac{\sigma }{r_{ij}}\right)}^{12}-{\left(\frac{\sigma }{r_{ij}}\right)}^{6}-{\left(\frac{\sigma }{r_{c}}\right)}^{12}+{\left(\frac{\sigma }{r_{c}}\right)}^{6}\right]$$

for $r_{ij}\leq r_{c}$ and zero otherwise. This truncated and shifted potential provides excluded volume for each bead, such that two beads do not occupy the same spatial location at the same moment. The variable $r_{ij}=\left|\vec{r_{i}}-\vec{r_{j}}\right|$ defines the Euclidean distance between two given beads i and j. A cutoff distance of $r_{c}=2^{1/6}\sigma$ limits computational demand. As usual, all quantities in this study will be expressed in terms of the size of the beads σ and their interaction strength ε.

Nearest neighbor interactions along the polymer chain have an additional potential energy as defined by the finitely extensible nonlinear elastic (FENE) potential:

(2) $$U_{\mathrm{FENE}}\left(r_{ij}\right)=-\frac{1}{2}\kappa R_{0}^{2}\ln\left[1-{\left(\frac{r_{ij}}{R_{0}}\right)}^{2}\right]\;\;\;for\;\;\;r_{ij}<R_{0}$$

This anharmonic potential diverges as $r_{ij}\;$ approaches $R_{0}$. In our simulations, $R_{0}=1.5$ and κ=30. This combination of parameter values provides attractive and repulsive potential energies and gives rise to polymer chains that are incapable of passing through one another. In order to model telechelic associating polymers, beads that terminate chains can be connected by this same (FENE) potential. This connection is reversible. Once every 20 LJ steps, an attempt is made to break or form these temporary bonds using a Monte Carlo (MC) step [23]. To this end, the energies of the system before and after potential bond updates are compared. A step in which the energy increases is accepted with a probability dictated by the Boltzmann factor $e^{-\left(U_{n\mathrm{ew}}-U_{\mathrm{old}}\right)/kT}$. Decreases in energy always result in acceptance. The bonded state has an additional energy of $U_{assoc}=-22,$ which accounts for the attractive interaction between associating beads. The temperature is controlled by coupling the system to a heat bath. This hybrid MD/MC algorithm was developed in our group [24], and has been used since by several other groups [25,26], to model associative polymers.

### 2.2. System Properties

Temperature-dependent, structural transitions in the simulation were shown to be due to a gelation process [24]. Fluid-like dynamics can be observed at high temperatures, transitioning to near solid-like dynamics at lower temperatures. The micelle transition, occurring near T=0.51, marks the crossover between these two significantly different material properties. This temperature is typically characterized as a structural transition, identifying the point where the functionalized end groups cluster together and form finite size micelles. The total number of temporary bonds and therefore their total contribution to the energy increases rapidly at the gel transition. Hence, this structural transition is accompanied by a peak in the specific heat. At even lower temperatures (T=0.3) the lifetime of the temporary bonds becomes so long that the system ceases to flow. All experiments, in this work, are performed at a temperature of T=0.4, below that of the structural micelle transition and above that of dynamic arrest.

The system contains 1000 polymer chains, each of which contains 8 beads. A total of 30% of the volume is occupied by these beads. Hence, glassy effects due to bead packing play no role. The system is confined by two solid surfaces, a distance of 26.4 separated from each other. A total of 5% of the polymer chains are connected with one end to these surfaces so as to avoid wall slip. A sinusoidal strain $\gamma =\gamma_{0}\sin\omega \tau$ is applied to the upper surface, while the force F necessary to maintain the motion is traced. The time-dependent stress σ (being different from the length scale of a bead) within the system can then be calculated as σ=F/A, where A = 58.8 is the cross-sectional area of the simulation cell. In these oscillatory rheology experiments, the system settles into a steady state after an initial transient regime, which lasts only a couple of cycles at small amplitude, but can be a few dozen of cycles at the largest amplitude studied. All data reported are obtained from the steady state regime. Transient phenomena are not discussed in this publication.

### 2.3. Network Topology

**Figure 1:**
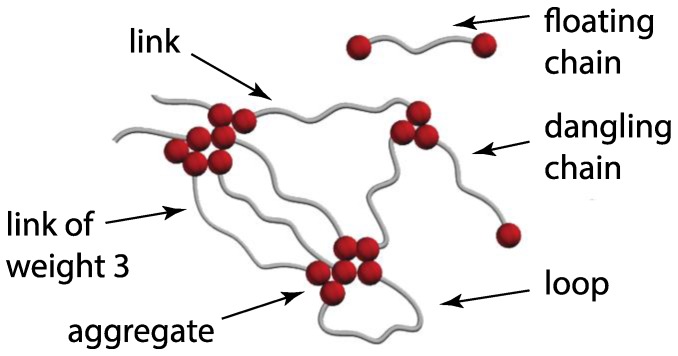
Illustration of possible network topologies. Bead-spring polymers contain 8 beads; those at the ends (red) attract each other. The beads on the gray chains are suppressed for clarity.

At the temperature of this study, the polymer chains form a network that changes over time. Several structural components of this network are illustrated in Figure 1. The associating groups (in red) tend to aggregate in small clusters. Polymers form either a loop (if both ends are part of the same aggregates) or a bridge (if they connect two different aggregates). Multiple chains can connect the same aggregates. We call this a link. Figure 1 illustrates a link of weight 3, where weight indicates the number of bridging chains. Floating and dangling chain configurations exist as well.

## 3. Results

### 3.1. Moduli

**Figure 2:**
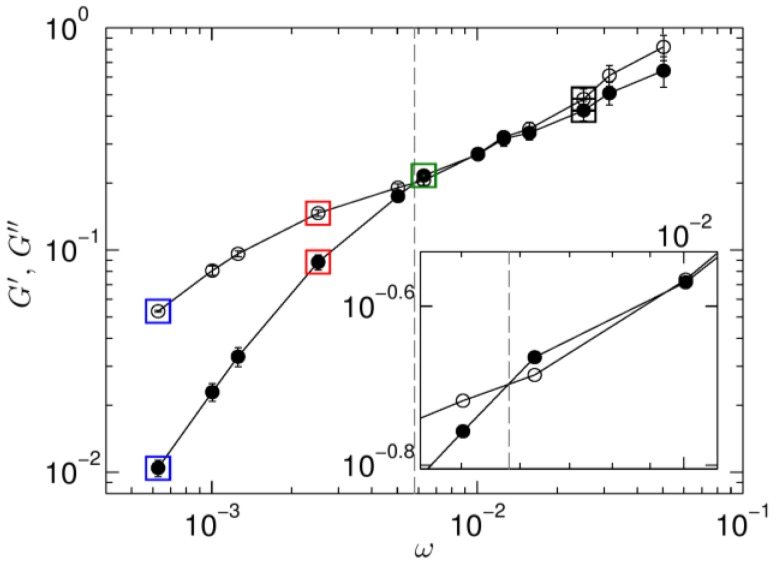
The frequency dependence of the loss (open symbols) and storage moduli (closed symbols) obtained at a strain rate of $\dot{\gamma }=2.26\;\times \;10^{-3}$. The crossover frequency where $G^{\prime \prime }=G^{\prime }$ is marked by a dashed gray line. Data are reported as the average of the primary harmonic as determined at each period of oscillation. Error bars are the standard deviation in this data. The inset shows a closer view near the crossover frequency. Four frequencies are identified within the frequency range as square symbols. The coloring scheme remains consistent throughout the manuscript.

The storage $G^{\prime }$ and loss moduli $G^{\prime \prime }$ are determined as the primary harmonic of a Fourier transform of the stress response at each period of oscillation. This result is then averaged over all cycles and reported as a function of oscillation frequency ω (Figure 2). All data are obtained at a constant maximum strain rate of ${\dot{\gamma }}_{max}=\gamma_{o}\omega =2.26\times 10^{-3}$. With increasing frequency, four regimes can be observed: a low frequency viscous regime ($G^{\prime \prime }>G^{\prime }$), a crossover frequency where $G^{\prime \prime }=G^{\prime }$, a short elastic regime $\left(G^{\prime \prime }<G^{\prime }\right)$, and finally a high frequency viscous regime. Microstructural studies are performed at four different frequencies, indicated by squares of four different colors in Figure 2. As we will show below, nonlinear behavior is most pronounced at two of them. At ω = 6.28 × 10^−4^ the strain amplitude equals 3.59 and nonlinearities result from such a large deformation. At ω = 6.28 × 10^−3^ the strain is much smaller (0.36) and elastic dominates the viscous response. Such a transition from viscous to elastic response as a result of an external force has been observed in many experimental studies as well [9].

### 3.2. Viscoelastic-Structural Response

**Figure 3:**
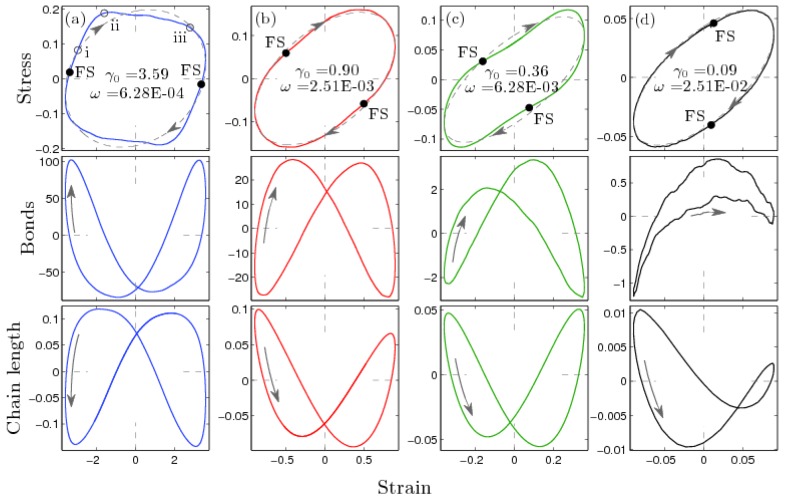
Lissajous trajectory curves are shown for four different frequency ω and amplitude γ_0_ combinations. Columns (**a**) through (**d**) contain data at the frequency and strain amplitude values reported within the center of each pane. The top row shows the stress-strain relationship. The stress σ as a function of the strain γ is shown as a colored line, with the linear contribution to these data shown as a dashed gray line. The middle row shows the strain dependence of the number of bonds. The average end-to-end chain length is shown in the bottom row. All data are averaged over multiple periods of oscillation. Arrows indicate the direction of the given trajectory in time. FS is the most favorable state, the one at which the number of temporary bond peaks and the chain length reaches a minimum.

The top panes of Figure 3 contain four Lissajous trajectory curves that span the frequency regimes mentioned above. The figures show the stress (colored lines) as a function of the applied strain. Each of the four plots is color coded to match the squares shown in Figure 2. The linear response of the stress (dashed gray lines) in each pane is calculated as the inverse Fourier transform of the primary harmonic to the stress response. Nonlinear characteristics can be observed as deviations of the trajectory curve from this ellipsoidal linear response. As seen in Figure 3, these trajectories show significant differences between their nonlinear contributions. Pane (a) occurs in the viscous regime. There is an observed overshoot in the stress as the strain approaches the zero-strain position. The trajectory in pane (b) has the opposite behavior, i.e., a slight undershoot in stress as the strain approaches its zero-strain position. The trajectory in pane (c) occurs at a frequency slightly above the observed crossover frequency. As seen in the inset of Figure 2, the moduli indicate that this response is elastic. The trajectory shows that the stress undershoots near the zero-strain position and overshoots close to the extreme in strain. The trajectory in pane (d) occurs in the high frequency regime. This trajectory is mostly linear, as evidenced by only small deviations from the dashed gray line.

In order to understand the origin of the nonlinear behavior, we investigate the time series of structural features contained within the polymer network. The deviation of the average number of bonds is reported in the middle panes of Figure 3, and the deviation in the average chain length (that is, end-to-end distance) is shown in the lower ones. For comparison, before the application of shear the average number of bonds is 5850 and chain length 4.11. Loop structures (see Figure 1) are excluded from the chain length calculations.

For each of the four frequencies reported, the number of bonds and the chain length are out-of-phase. However, their values peak at different moments during the oscillations. We consider a maximum in the quantity of bonds, with a minimum in chain length, to be the most favorable state (FS). As the upper surface continues through the cycle, chains stretch and bonds break. Ultimately, the system reaches the most unfavorable state (UFS). We now will look at this process in more detail. In what follows, we will concentrate on the two most nonlinear cases: γ_0_ = 3.59; ω = 6.28 × 10^−4^ (type I) and γ_0_ = 0.36; ω = 6.28 × 10^−3^ (type II).

At ω = 6.28 × 10^−3^ (type II shown in Figure 3c), the FS occurs slightly before the zero-strain position of the upper surface. Moving away from the FS, chains become stretched as strain is applied. This results in bond stretching and ultimately bond breaking. Once the upper wall reaches the extreme (UFS) and starts to move back towards the zero-strain position, healing takes place. Overshoot in stress occurs during the healing events. An inflection point is observed in the stress slightly before the maximum in the number of bonds. This is followed by a breaking of bonds and an undershoot in the stress.

At ω = 6.28 × 10^−4^ (type I shown in Figure 3a), different behavior is observed. Note that this trajectory occurs at the large strain amplitude (3.59). The relationship between bonds and chain length remains the same, but the maximum number of bonds (FS) is now reached just after the upper wall moved through its extreme, whereas the UFS occurs near its zero-strain position. During movement from FS to UFS, stress overshoots. During the healing process, when the system moves from UFS to FS, it undershoots. The point of inflection in the stress correlates well in time to the minimum in bonds (UFS). All of this is opposite to the earlier results at ω = 6.28 × 10^−3^.

For these two frequencies, nonlinearity results from the fact that the microscopic changes of the temporary topological network structure are not fast enough to keep the system at equilibrium. As a result, the stress overshoots or undershoots as compared to the linear response. The data in the lower two panels of Figure 3 show that even when at the same strain, the system is in structurally distinct states according to the number of bonds and chain length when moving towards or away from the zero-strain position. The observed hysteresis confirms that microstructure of a telechelic polymer system depends on the history of deformation when subject to LAOS shear.

### 3.3. Topological Features

**Figure 4:**
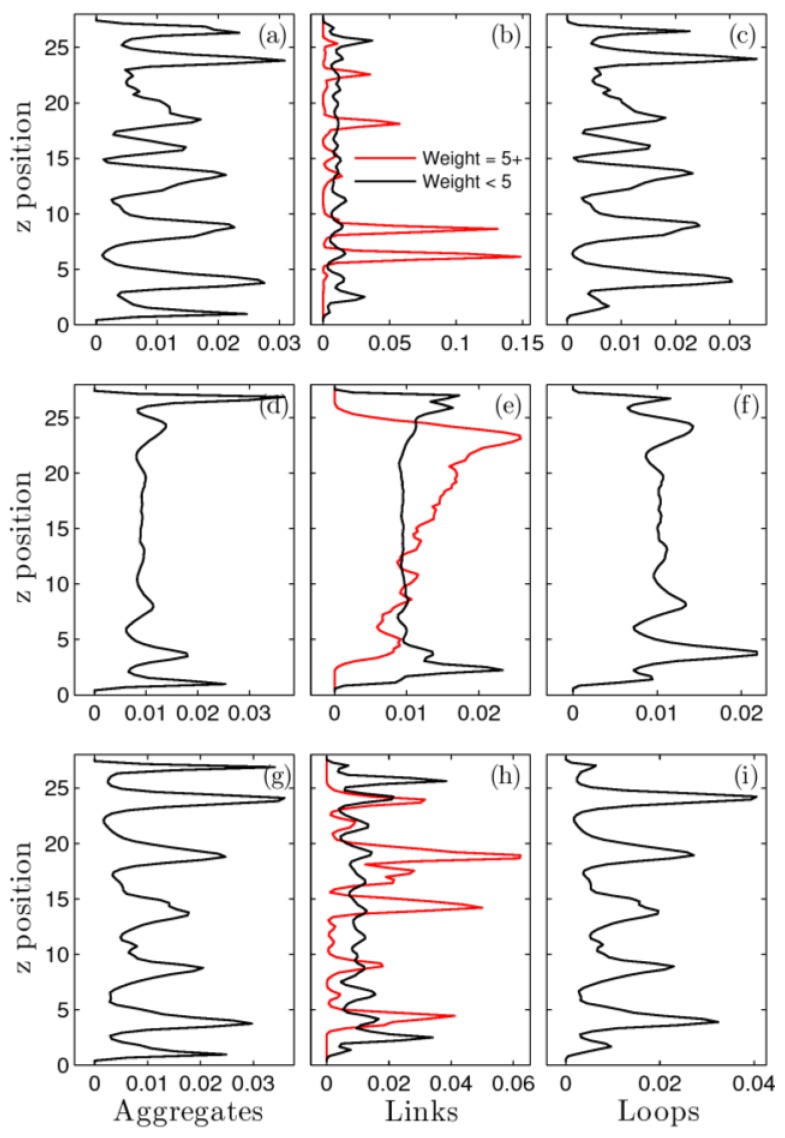
Topological features for (**a**–**c**) system at rest; (**d**–**f**) $\omega =6.28\;\times \;10^{-4}$, γ_0_ = 3.59; and (**g**–**i**) $\omega =6.28\;\times \;10^{-3}$, $\gamma_{0}=0.36$. The position of aggregates, links, and loops is shown as a probability distribution as a function of the distance (z) to the bottom of the film. Values are averaged over many oscillatory cycles. Link data are split in those for links less than weight five (black) and those for weight five and higher (red).

Next, we investigated how the network topology of the nonlinear non-equilibrium steady state differs from that of the the system at rest. This is shown in Figure 4. All data are statistical averages for the entire system over many oscillations after steady-state is reached. Figure 4a–c shows features of the system at rest. These data are compared to those for the system under type I, large amplitude oscillatory shear at $\omega =6.28\;\times \;10^{-4}$ (d–f) and the higher frequency, type II oscillation at $\omega =6.28\;\times \;10^{-3}$ (g–i). Figure 4a,d,g show the position of the aggregates. As we reported before [18,27], the system forms layers at this temperature, which causes the peaks and valleys in the aggregate distribution as a function of the distance from the bottom surface (z) in Figure 4a. Under type I oscillatory shear (middle panel) layering disappears, however it is observed under type II (lower panel). Figure 4b,e,h shows the position of the middle of links. Links that contain less than five chains and those that contain five or more are reported independently.

Figure 4c,f,i shows the position of loops, defined as the average position of the two end beads of the chains that form a loop. The most significant feature is the distribution of the high weight links (red line in Figure 4e). They seem to prefer the upper wall. We will come back to this phenomenon in Section 3.6. The topological structure of the type II system resembles that of the system at rest. Other topological differences between a system at rest and under shear were pointed out in our previous publications [18,27]. Among others, we reported an increase in the average aggregate size caused by shear. A novel phenomenon found in this previous work is the increase in the weight of links as a result of shear: the system responds to external stress and restructures its topology so as to minimize dissipation. It does so by forming fewer but stronger links containing more polymers. The resulting sparser network is easier to deform.

### 3.4. Aggregate Dynamics

**Figure 5:**
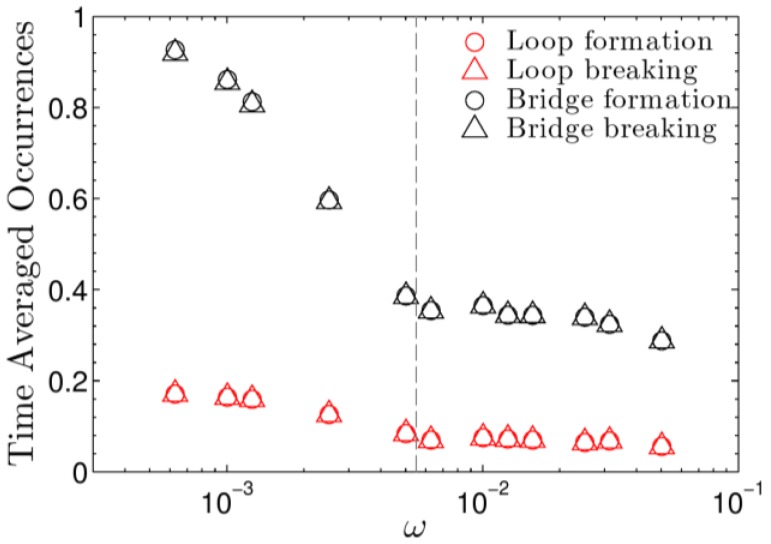
The time averaged number of loop and bridge aggregation events. The crossover frequency is indicated by a dashed line.

Next, we focus on the dynamics the system, which, as we will show, is very much dependent on the oscillation frequency. Over the given frequency spectrum, we trace the number of aggregation events, separating events that involve loops and those involving bridges (see Figure 1 for definitions). Figure 5 shows the time-averaged number of occurrences as a function of oscillatory frequency. Two different trends can be observed within the data. For both the loop and bridge data, the lower frequency regime exhibits a steeper slope than the high frequency regime. The change in slope occurs near the observed crossover frequency defined in Figure 2. The number of bridge breaking and formation events is observed and expected to be larger than that of loops. This is due to the stresses induced from the stretching of bridging chains. Chain stretching results in associating groups being freed from their corresponding aggregates; this relaxes stress. Loops do not encounter stresses imposed by chain stretching. They primarily contribute to the frictional component of stresses. As expected, after averaging over many oscillations, temporary bonds break and form at the same rate. Consequently, the loop to bridge ratio stays the same.

**Figure 6:**
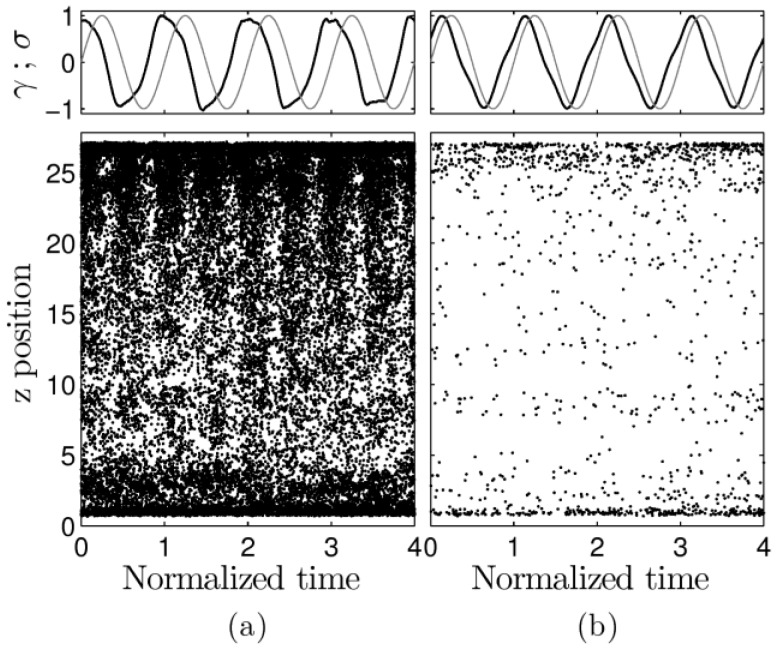
Stress (black)-strain (gray) relationship (top row) and spatial-temporal plots of aggregate formation (lower row) as a function of normalized time t/T, where *T* is the period of the oscillation. Each point in the spatial-temporal plots locates the formation of an aggregate within time. Panes in (**a**) correspond to the frequency-amplitude combinations $\omega =6.28\;\times \;10^{-4}$, γ_0_ = 3.59; and (**b**) $\omega =6.28\;\times \;10^{-3}$, γ_0_ = 0.36.

As we showed above, within an individual oscillation there are timespans during which bond breaking dominates and others during which bonds tend to restore. Figure 6 contains spatial-temporal plots, reporting the z-position of aggregate formation in the simulation cell. Data are shown for the two most nonlinear trajectories: type I in (a) and type II in (b). The variations in stress (black) and strain (gray) are shown in the top panes. The lower panel shows a black dot for each bond-forming event. Data obtained for the breaking of aggregates produce results similar to those observed in these plots (not shown). The system is highly dynamic during large amplitude oscillations, much more so than during type II deformation. In both cases, events occur throughout the system but are more frequent close to the walls. For the large amplitude oscillations, the density of activity in time is seen to follow a cycle at twice the frequency of strain oscillation.

### 3.5. Repetitive Structures

**Figure 7:**
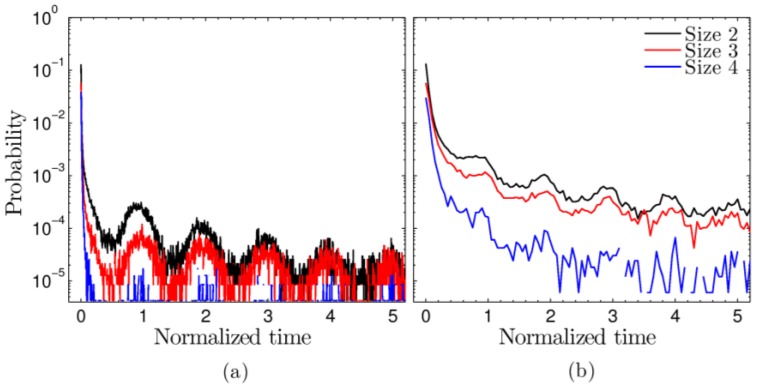
Probability of a given time span between the break of an aggregate and the cyclic formation of the same aggregate. Data are shown for frequency-amplitude combinations (**a**) $\omega =6.28\;\times \;10^{-4}$, γ_0_ = 3.59; and (**b**) $\omega =6.28\;\times \;10^{-3}$, γ_0_ = 0.36. Time is normalized by the period of an oscillation.

In the previous sections we have shown that even though structural and dynamic oscillations in the microstructure occur during each oscillation of the upper wall, the topological features of the network do not change after many oscillations. We wonder if only the overall statistics of the topological structure remain the same or if actually the same associating groups form the aggregates—that is, if structures are formed repetitively. If so, this would imply a structural memory within the system. To this end, we trace each aggregate that breaks, labeling the aggregate as the collection of its specific associating groups. Later in time, when an aggregate forms, we search the list of broken aggregates in an effort to identify whether the system has formed a repetitive structure. Note that if the same aggregate restores multiple times, it is counted each time. We refer to such an aggregation event as a cyclic reaction. We are interested in the time span between the original breaking and the subsequent formation. Figure 7 shows the probability of this span for cyclic aggregation events. Data are shown for the same two frequencies explored earlier. Most of the aggregates that restore do so immediately. However, as seen in the figures, this is not always the case. Cyclic events are quite common and are more likely to occur after one or more full oscillations of the system. The figures show data for three aggregate sizes. For each of the frequencies, the smaller the aggregate, the more likely that it restores. In the higher frequency type II oscillations (b), cyclic events can last very long: the system reforms the same aggregate over and over for upwards to 50 oscillations (not shown), whereas, for large amplitude type I oscillatory shear (a), the likelihood of reformation drops faster. However, in this case, the increased probability that a cyclic event occurs after full period time spans is more pronounced. In studies of sheared jammed solids [28], it has likewise been found that systems often fall back into a configuration that had already been visited in a previous cycle, despite undergoing many particle rearrangements during the cycle. Hence, such periodic states could be general phenomena of complex systems near a jamming, glass, or gel transition. Their existence may be indicative of such a transition.

### 3.6. LAOS and Shear Banding

**Figure 8:**
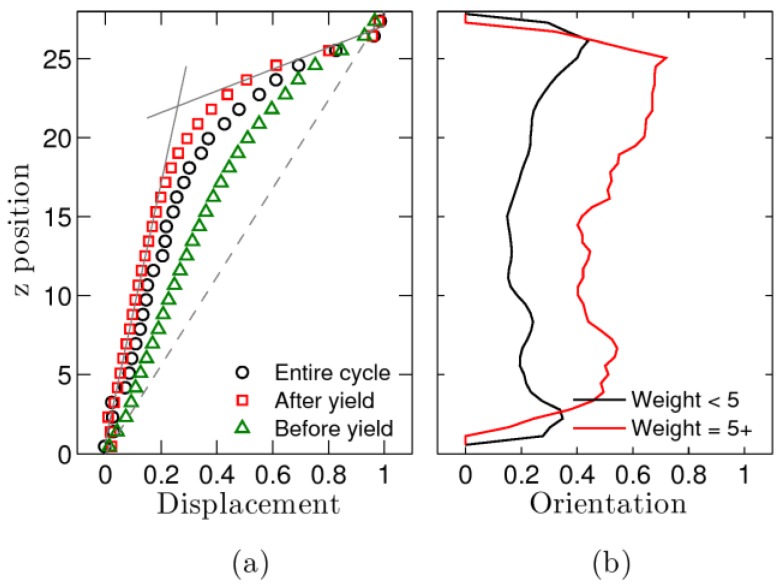
(**a**) Displacement of a layer at a position z from the bottom of the film as a fraction of the displacement of the upper wall. The black circles represent averages over the entire cycle, the red squares those after yield, and the green triangles those before yield. (**b**) Orientation Q_xx_ in the shear direction of the polymer chains that link the aggregates. Data are split in those for links less than weight five (black) and those for weight five and higher (red). See text for discussion.

Type I large amplitude oscillatory shear simulations are investigated in more detail. As we reported earlier, the system reaches its favorable state (FS) near the extreme. The number of temporary bonds is at a maximum and chain length at a minimum. When moving from one extreme to the next, stress initially overshoots and then undershoots. In Figure 3a, the point at which nonlinear terms start to contribute to the stress is indicated by (i), the point at which they stop being important by (iii), and (ii) indicates the moment at which the stress reaches a maximum. Further analysis indicates that at this moment the system yields and starts shear banding. This can be seen in Figure 8a. The relative displacement of the polymer beads compared to that of the upper wall is measured as a function of the distance (z) from the bottom surface. The black circles result from averages over the entire oscillation. They indicate that the shear profile is not uniform, since they deviate from the dashed line. Green triangles and red squares measure the displacement during the (i) to (ii) and (ii) to (iii) timespans, respectively. The green triangles follow a smooth curve; the red square curve has a kink. Its displacement profile looks similar to that observed for systems under uniform shear that exhibit banding [27,29]. A small layer near the upper wall moves faster than the remainder of the film. As in our previous work [27], we fit the data with two straight lines. The point at which they cross corresponds with the interface between the shear bands.

Shear banding has been observed for telechelic polymers, but most studies were performed in micellar systems [30,31]. Structural differences between shear bands were observed there. In particular, an increase in nematic order in the shear-band near the moving surface was detected. We observe a similar increase in orientational order in our simulations of associating polymers. This is shown in Figure 8b and measured by the following quantity:

(3) $$Q_{\mathrm{xx}}=\frac{3}{2}\left[{\left(\frac{x}{r}\right)}^{2}-\frac{1}{3}\right].$$

Here, *r* is the end-to-end distance of a polymer and *x* its component in the x direction (the direction of imposed shear). For randomly oriented polymers *Q*_xx_ = 0. Due to the applied shear one expects it to be positive. The data show that *Q*_xx_ is larger near the upper wall. The red curve shows the data for polymers that are part of links containing five or more chains. For these, the effect is even more pronounced. As shown in Figure 4e, the higher weight links tend to locate in the upper shear band. We conclude that the microscopic changes that occur during each movement, from one extreme to the other, resemble those occurring under uniform shear. Stress builds up, this is then followed by a yield event, shear banding, and flow. Ultimately, the system heals when reaching the other extreme.

## 4. Discussion and Conclusions

Nonlinear response of associating polymer networks is studied by means of large-scale computer simulations. The system is confined between two surfaces and an oscillatory shear is applied to the upper one. Lissajous figures indicate that there are two frequency-amplitude regimes for which the response is highly nonlinear: a low frequency—large amplitude (type I) and smaller amplitude—higher frequency (type II) combination. Type I occurs at a strain amplitude much larger than one. Type II occurs at a frequency at which the elastic storage modulus starts to dominate the viscous loss modulus. For intermediate frequencies and amplitudes, the response is much closer to linear. The simulations reveal the microstructural origin of the nonlinearities, which is different in both cases. The most favorable (FS) state—the one in which polymer chains are relaxed and the number of temporary bonds peaks—is reached near the extreme in type I, and closer to the zero-strain position in type II. The network topology in type II stays closer to that of the initial unsheared system, whereas, in type I, the breaking and formation of temporary bonds are less frequent than in type II. Most interestingly, during large amplitude oscillations, the system tends to reform bonds between the same associating groups over and over again, for up to dozens of cycles, even though the system undergoes major restructuring. During each cycle, the network yields twice. This behavior is very similar to that observed under uniform shear [27]. While moving from one extreme to the other, the stress increases and yield occurs. This is followed by shear banding. Like in the studies of systems under uniform shear deformation, the network reconfigures itself so it can more easily deal with large deformations; the number of links between aggregates is reduced by having multiple polymers link in between the same aggregates. A sparser network results.

The simulations in this work were performed using a short bead-spring toy model. Nevertheless, we believe that the internal microscopic changes that give rise to the non-equilibrium mechanical behavior in this study are similar to those that underpin the nonlinearities reported in simulations and experiments of complex systems performed by other groups. Type II deformations were reported in filled rubbers [32] and other experiments of polymer nanocomposites [33,34,35]. In these materials, the fillers function as temporary crosslinks between polymer chains. Several studies of emulsion show type II deformations as well [36,37]. Fewer studies were performed at large amplitude oscillatory shear. Nonetheless, Hyun et al. [38] observed type I behavior in their experiments on polymer nanocomposites. In addition, Swan et al. [39] conducted large amplitude oscillatory microrheology of colloidal suspensions. In one regime, they observe type I behavior, which they believe is caused by a tendency of the microstructure to minimize dissipation by opening up a channel through which their probe particle can move. The resulting nonlinear, microstructural deformation and the ability of the system to store it in its memory causes the type I behavior. Even though colloidal systems and associating polymers appear to be quite different, they are “similar” in the sense that the connectivity between end groups in gel polymer networks plays a similar role as packing rearrangements in glassy jamming colloidal dispersions. In a polymer gel, flow is restricted by the associating bonds, while in a glass the absence of free volume hinders motion. The associative polymeric system at hand restructures to form a sparser network under large amplitude shear, while colloidal suspensions rearrange in such a way that a channel forms. We have pointed out the similarities between the gel and glass transition in a previous publication [24].

As pointed out above, type I behavior is also observed in wormlike micellar systems [31,40,41]. Since their dynamics likewise include scission and recombination events, these systems are closely related to associating polymeric systems. Shear banding, yielding, and alignment sequences, very similar to those that we describe in this study of associating polymers, are observed. In particular, an increase in the orientational order parameter in the fast moving shear band has been reported, a phenomenon that we believe is similar to the one we show in Figure 8. As we mentioned in the introduction, based on these observations, these authors [17] have pointed out the shortcomings of older theoretical frameworks for LAOS. Using a rheo-SANS approach, they observe that indeed sequences of physical processes dictate the microstructure and hence the stress response. Our simulations confirm these observations for associating polymer systems.

To improve our current theoretical models of soft complex fluids under large oscillatory shear, we need to better understand how information of previous deformations is encoded in their microstructure. This memory could be stored spatially in features such as chain extension, the location of associative bonds, or dynamically through the creation of periodic states. Our future studies will explore this last possibility in more depth. Moreover, we intend to use more realistic force fields than the toy model applied in the present one. In particular, we will focus on a model of mucus—an associative polymer network that protects the human body.

## References

[1] Hao J.K., Weiss R.A. (2011). Viscoelastic and Mechanical Behavior of Hydrophobically Modified Hydrogels. Macromolecules.

[2] Kujawa P., Wanatabe H., Tanaka F., Winnink F.M. (2005). Amphiphilic Telechelic Poly (*N*-Isopropylacrylamide) in Water: From Micelles to Gels. Eur. Phys. J. E.

[3] Sing M.K., Wang Z.-G., McKinley G.H., Olsen B.D. (2014). Celebrating Soft Matter’s 10th Anniversary: Chain Configuration and Rate-Dependent Mechanical Properties in Transient Networks. Soft Matter.

[4] Verso F.L., Likos C.N. (2008). End-Functionalized Polymers: Versatile Building Blocks for Soft Materials. Polymer.

[5] Glass J.E. (1996). Hydrophilic Polymers: Performance with Environmental Acceptability. Advances in Chemistry Series.

[6] Dhandayuthapani B., Yoshida Y., Maekawa T., Kumar D.S. (2011). Polymeric scaffolds in tissue engineering Application: A review. Int. J. Political Sci..

[7] Wojtecky R.J., Meador M.A., Rowan S.J. (2011). Using the Dynamic Bond to Access Macroscopically Responsive Structurally Dynamic Polymers. Nat. Mater..

[8] Zhuang S., Bassett D.S., Winkelstein B.A. (2016). Stretched-Induces Network Reconfiguration of Collegen Fibres in the Human Facet Capsular Ligament. J. R. Soc. Interface.

[9] Hawke L.G.D., Amandi M., Goldansaz H., van Ryumbeke E. (2016). Viscoelastic properties of linear associating poly (N-Butylacrylate) Chains. J. Rheol..

[10] Zinn T., Willner L., Lund R. (2016). Telechelic Polymer Hydrogels: Relation between Microscopic Dynamics and Macroscopic Viscoelastic Response. ACS Macro Lett..

[11] Semenov A.M., Rubinstein N. (1998). Thermoreversible Gelations in Solutions of Associating Polymers. Macromolecules.

[12] Witten T.A. (1988). Associating Polymers and Shear Thickening. J. Phys..

[13] Cho K.S., Hyun K., Ahn K.H., Lee S.J. (2005). A Geometrical Interpretation of Large Amplitude Oscillatory Shear Response. J. Rheol..

[14] Ewoldt R.H., Hosoi A.E., McKinley G.H. (2008). New Measures for Characterizing Nonlinear Viscoelasticity in Large Amplitude Oscillatory Shear. J. Rheol..

[15] Rogers S.A., Erwin B.M., Vlassopoulos D., Cloitre M. (2011). A Sequence of Physical Processes Determined and Quantified in LAOS: Application to a Yield Stress Fluid. J. Rheol..

[16] Rogers S.A., Erwin B.M., Vlassopoulos D., Cloitre M. (2011). Oscillatory Yielding of a Colloidal Star Glass. J. Rheol..

[17] Rogers S.A., Lettinga M.P. (2012). A Sequence of Physical Processes Determined and Quantified in Large-Amplitude Oscillatory Shear (LAOS): Application to Theoretical Nonlinear Models. J. Rheol..

[18] Wilson M., Rabinovitch A., Baljon A.R.C. (2015). Computational Study of the Structure and Rheological Properties of Self-Associating Polymer Networks. Macromolecules.

[19] Hyun K., Wilhelm M., Klein C.O., Cho K.S., Nam J.G., Ahn K.H., Lee S.J., Ewoldt R.H., McKinly G.H. (2011). A Review of Nonlinear Oscillatory Shear Tests: Analysis and Application of Large Amplitude Oscillatory Shear (LAOS). Prog. Polym. Sci..

[20] Wyss H.M., Miyazaki K., Mattsson J., Hu Z., Reichman D.R., Weitz D.A. (2007). Strain-Rate Frequency Superposition: A Rheological Prope of Superposition in Soft Materials. Phys. Rev. Lett..

[21] Hess A., Aksel N. (2011). Yielding and Structural Relaxation in Soft Materials: Evaluation of Strain-Rate Frequency Superposition Data by the Stress Decomposition Method. Phys. Rev. E.

[22] Kremer K., Grest G.S. (1990). Dynamics of Entangled Linear Polymer Melts: A Molecular-Dynamics Simulation. J. Chem. Phys..

[23] Baljon A.R.C., Depuy T., Vorselaars J. (2004). Computational Studies of Contact Time Dependence of Adhesive Energy due to Redistribution of the Locations of Strong Specific Interfacial Interactions. Macromolecules.

[24] Baljon A.R.C., Flynn D., Krawzsenek D. (2007). Numerical Study of the Gel Transition in Reversible Associating Polymers. J. Chem. Phys..

[25] Hoy R.S., Fredrickson G.H. (2009). Thermoreversible Associating Polymer Networks: I. Interplay of Thermodynamics, Chemical Kinetics, and Polymer physics. J. Chem. Phys..

[26] Amin D., Likhtman A.E., Wang Z. (2016). Dynamics in Supramolecular Polymer Networks Formed by Associating Telechelic Chains. Macromolecules.

[27] Billen J., Wilson M., Baljon A.R.C. (2015). Shear Banding in Simulated Telechelic Polymers. Chem. Phys..

[28] Lavrentovich M.O., Liu A.J., Nagel S.R. (2017). Period Proliferation in Periodic States in Cyclically Sheared Jammed Solids. Phys. Rev. E.

[29] Sprakel J., Spruijt E., Cohen Stuart M.A., Besseling N.A.M., Lettinga M.P., van der Gucht J. (2008). Shear Banding and Rheochaos in Associative Polymer Networks. Soft Matter.

[30] Manneville S., Colin A., Waton G., Schosseler F. (2007). Wall slip, Shear Banding, and Instability in the Flow of a Triblock Copolymer Micellar Solution. Phys. Rev. E.

[31] Lerouge S., Decruppe J.-P., Olmsted P. (2004). Birefringence Banding in a Micellar Solution or the Complexity of Heterogeneous Flows. Langmuir.

[32] Mermet-Guyennet M.R.B., Gianfelice de Castro J., Habibi M., Martzel N., Denn M.M., Bonn D. (2015). LAOS: The Strain Softening/Strain Hardening Paradox. J. Rheol..

[33] Lim H.T., Ahn K.H., Hyun K. (2013). Nonlinear Viscoelasticity of Nanocomposites under Large Oscillatory Shear Flow. J. Rheol..

[34] Papon A., Maribia S., Guy L., Mpntes H., Sotta P., Long D. (2012). Unique Non-Linear Behavior of Nanofilled Elastomers: From the Onset of Strain Softening to Large Amplitude Shear Deformations. Macromolecules.

[35] Li S., Mi Y., Wang X. (2017). Superposed Nonlinear Rheological Behavior of Filled Elastomers. J. Rheol..

[36] Ewoldt R.H., Winter P., Maxey J., McKinley G.H. (2010). Large Amplitude Oscillatory Shear of Pseudoplastic and Elastoviscoplastic Materials. Rheol. Acta.

[37] Brian M.E. (2010). Examing the Validity of Strain-Rate Frequency Superposition when Measuring the Linear Viscoelastic Properties of Soft Materuals. J. Rheol..

[38] Hyun K., Lim H.T., Ahn K.H. (2012). Nonlinear Response of Polypropylene (PP)/Clay Nanocomposites under Dynamic Oscillatory Shear Flow. Korea-Aust. Rheol. J..

[39] Swan J.W., Zia R.N., Brady J.F. (2014). Large Amplitude Oscillatory Microrheology. J. Rheol..

[40] Rogers S., Kohlbrecher J., Lettinga M.P. (2012). The Molecular Origin of Stress Generation in Worm-Like Micelles, Using a Rheo-SANS LAOS Approach. Soft Matter.

[41] Calabrese M.A., Wagner N.J., Dreiss C., Feng Y. (2017). Wormlike Micelles: Systems, Characterization and Applications.

